# Are MXenes viable as conductive, transparent films for industrial applications?

**DOI:** 10.1080/14686996.2025.2597556

**Published:** 2025-12-12

**Authors:** Tiezhu Guo, Shungui Deng, Rene Schneider, Chuanfang Zhang, Jakob Heier

**Affiliations:** a Functional Nanomaterials, Catalonia Institute for Energy Research (IREC), Barcelona, Spain; b Laboratory for Functional Polymers, Empa, Swiss Federal Laboratories for Materials Science and Technology, Dübendorf, Switzerland

**Keywords:** Transparent conductive electrodes, MXenes, figure of merit, percolation, theoretical limitation

## Abstract

Two-dimensional transition metal carbides and nitrides, so-called MXenes, hold significant promise as flexible transparent conductive electrodes (TCEs) in diverse applications. However, MXenes fall below the minimum requirements for industrial use, largely due to factors such as the quality of MXene flakes, electrical conductivity, optical conductivity, and transparent electrode fabrication techniques. In this study, we analyze the relationships among nanosheet size, DC- and optical conductivity and its ratio ($\sigma_{\mathrm{DC}}$/$\sigma_{\mathrm{op}}$), and sheet resistance (*R*_s_) of MXene TCEs based on data from published literature. Compared to Ti_3_C_2_T_*x*_ TCEs fabricated with low-quality, small-sized flakes ( <1 μm, $\sigma_{\mathrm{DC}}$/$\sigma_{\mathrm{op}}$ < 10), those made with high-quality, large-sized nanosheets ( >6 μm, $\sigma_{\mathrm{DC}}$/$\sigma_{\mathrm{op}}$ > 20) with narrow size distributions exhibit dramatically reduced *R*_s_ by several orders of magnitude while maintaining high transmittance. Nevertheless, the $\sigma_{\mathrm{DC}}$/$\sigma_{\mathrm{op}}$ of continuous Ti_3_C_2_T_*x*_ metallic TCEs saturates at ~24, fairly below the basic requirements for commercial TCEs. By integrating a metallic silver grid onto Ti_3_C_2_T_*x*_ TCEs, a remarkable $\sigma_{\mathrm{DC}}$/$\sigma_{\mathrm{op}}$ ratio of 330 has been achieved, bringing MXene TCEs closer to fulfilling industrial application standards and inspiring greater confidence in their future adoption. Beyond the field of TCEs, the insights gained here could inspire advancements in other areas, such as optoelectronic devices, flexible displays, and energy-efficient transparent technologies. This work provides a framework for the design and development of next-generation transparent conductive materials with broad implications across various scientific and industrial domains.

## IMPACT STATEMENT

A coated MXene film alone cannot meet industrial standards for transparent conductive electrodes; however, when integrated with a printed silver grid, it successfully achieves the required transparency and conductivity levels.

## GRAPHICAL ABSTRACT

**Figure:**
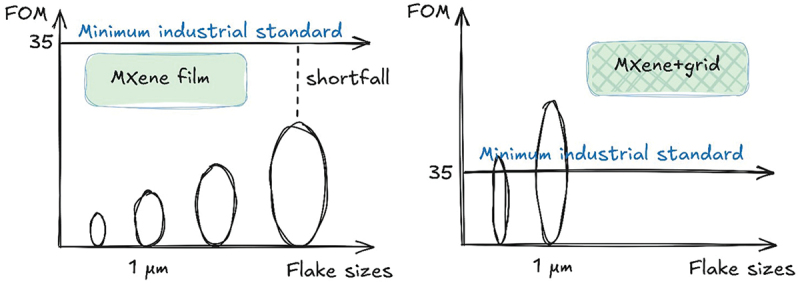

## Introduction

Transparent conductive electrodes (TCEs) with high conductivity and transparency are essential for a wide range of applications, including transparent supercapacitors, antennas, electromagnetic shielding, and sensors, among others [1]. Traditionally, indium tin oxide (ITO) and fluorine-doped tin oxide (FTO) have served as the first generation of commercial TCEs, offering low sheet resistance (*R*_*s*_, < 10 Ω sq^−1^) and high transparency (*T*, > 90%) in the visible range [1–4]. However, as ceramic materials, ITO and FTO are prone to cracking and fracturing under relatively low strains of 2 ~ 3% [5]. The expansion of microcracks further results in a sharp increase in resistance. This limitation has driven the development of flexible transparent electrodes as replacements for ITO in flexible electronics.

Promising alternatives for flexible TCEs include transparent conductive films based on materials such as silver nanowires, carbon nanotubes (CNTs), PEDOT, graphene, and MXene, but also meshes fabricated from metals [3,4,6,7]. The performance of TCEs is typically characterized by the relationship between transparency (*T*) and sheet resistance (*R*_s_), as described by the following equation [8]:

(1) $$T=(1+\frac{188.5}{R_{s}}\frac{\sigma_{\mathrm{op}}}{\sigma_{\mathrm{DC}}}{)}^{-2}\;$$

where $\sigma_{\mathrm{op}}$ is optical conductivity, $\sigma_{\mathrm{DC}}$ is DC conductivity, $\sigma_{\mathrm{DC}}$/$\sigma_{\mathrm{op}}$ is defined as the figure of merit (FOM_e_), which is commonly used to evaluate the optoelectronic performance of TCEs. A higher FOM_e_ indicates that films can be thinning down to the highly transparent region without dramatically increasing its resistance. To replace fragile ITO in many applications, the minimum industrial standard must be met, which requires *R*_*s*_ < 100 Ω sq^−1^ at *T* > 90% in the visible range [1]. This corresponds to a basic requirement of $\sigma_{\mathrm{DC}}$/$\sigma_{\mathrm{op}}$ > 35, as derived from Equation 1 [1]. However, many cases demand *R*_*s*_ < 10 Ω sq^−1^ at *T* > 85%, implying that $\sigma_{\mathrm{DC}}$/$\sigma_{\mathrm{op}}$ > 220 must be achieved [1].

We note that the upper limits of the optoelectronic properties of graphene-based TCEs have been discussed in previous studies, with maximum figure of merit ($\sigma_{\mathrm{DC}}$/$\sigma_{\mathrm{op}}$) values saturate at ~0.7 and 11 for liquid-exfoliated and CVD graphene flakes (without doping), respectively [8]. Clearly, these values fall significantly below the minimum industrial standards. MXene, a family of 2D transition metal carbides, nitrides, and carbonitrides, offers excellent conductivity and being highly transparent when the thickness is several nanometers. Moreover, MXene is water-dispersible and can be processed into transparent conductive films at room temperature using methods such as blade coating, slot-die coating, and spin coating [3,4,9]. Some Ti_3_C_2_T_*x*_-based TCEs have demonstrated impressive optoelectronic performance, such as a sheet resistance of 222 Ω sq^−1^ with a transmittance of 92% [4]. However, these results remain insufficient to replace ITO or silver nanowires as TCE materials.

We also note that to date, the true metrics for evaluating MXene-based transparent electrodes have yet to be established. For instance, most studies only report the transmittance (*T*) and sheet resistance (*R*_s_) of the films, which are often inconsistent due to varying film thicknesses. This variability makes direct comparisons between MXene-based films and other TCE materials difficult. At high transmittance (*T* > 90%), even slight changes in transparency can lead to a sharp increase in *R*_s_, as the reduced thickness compromises conductive pathways within the electrode. Utilizing the figure of merit (FOM_e_) to represent the optoelectronic properties relative to other conductive transparent films remains the most straightforward parameter for comparison. Additionally, we recognize the importance of understanding the upper limit of FOM_e_ in MXene TCEs, as it tells us the maximum achievable value and the potential in practical applications. However, to date, no reports on the theoretical limitation of FOM_e_ are available for MXene TCEs, to the best of our knowledge.

In this work, we analyze the relationships between MXene nanosheet size, average conductivity, type of MXene (e.g. Ti_3_C_2_T_*x*_, Ti_2_CT_*x*_, V_2_CT_*x*_, Ti_3_CNT_*x*_, etc.), figure of merit, optical conductivity, and sheet resistance in TCEs at high transparency. We identify the general trends and key factors affecting the optoelectronic performance of TCEs (e.g. the relationship between *T* and *R*_s_), revealing the potential maximum achievable FOM_e_ fairly below the minimum industrial requirements. As such, we introduce a metallic Ag mesh onto the MXene TCEs, leading to significantly reduced *R*_*s*_ while marginally affecting *T*. The Ag mesh-MXene TCEs exhibit a record-high FOM_e_ up to 330, positioning MXene as a strong candidate for use as a flexible transparent electrode in industrial applications.

## Experiments

### Fabrication of MXene nanosheets

0.8 g of LiF was added to 10 ml of 9 M HCl and stirred continuously in an oil bath at 35°C for 10 minutes. Then, 0.5 g of Ti_3_AlC_2_ was gradually added to the above solution. After 24 hours of reaction, the resulting precipitate was washed with deionized water and centrifuged at 1,500 rcf for 5 min until the supernatant became dark green and the pH approached ~6. Next, 100 ml of deionized water was added to the precipitate and manually shaked until the precipitate was completely re-dispersed. The solution was degased under argon for 10 min, then sonicated continuously in an ice bath for 60 min at 35 kHz. The dispersion was centrifuged at 1,500 rcf for 30 min and the upper suspension was collected to obtain Ti_3_C_2_T_*x*_ nanosheets.

### TCE fabrication

The silver meshes were printed through an aerosol jet printer (AJ-5X, Optomec, US) with a nozzle of size 100 μm using the Novacentrix JS-ADEV N250 silver nanoparticle ink on top of the MXene film. During the printing process the ink flow was kept constant, so that the variation of the printing speed (6, 9 and 12 mm/s) controls the printed line width and height, respectively. The uniform Ti_3_C_2_T_*x*_ films were coated onto glass substrate using the slot-die coating method, with the fabrication process and characterization details of the MXene nanosheets provided in our previous study [3]. After drying the ink at room temperature, the samples were annealed at 200°C on a hot plate under argon atmosphere for 2 hours.

### Film characterization

The topography of the printed meshes was obtained by confocal microscopy. The microscope (DCM8, Leica, Germany) was equipped with a EPI-150X-L objective from Leica, which offers a resolution of 140 nm and 2 nm in x,y- and z-direction, respectively. The sheet resistance (*R*_s_) of the Ag/MXene films was measured using a four-wire measurement method (2400 SourceMeter, Keithley, US) under ambient conditions. For the MXene films, measurements were conducted using a RM3-AR four-point probe (Jandel, UK), with the final value calculated as the average from 10 independent locations on sample. Optical transmittance spectra were recorded using a UV-vis spectrophotometer (Varian Cary 50, Agilent, US) over the wavelength range of 350–800 nm. The transmittance at 550 nm, a commonly used reference wavelength for evaluating the transparency of thin films, was determined as the average of five measurements taken from randomly selected positions on each TCE sample. In this study, the FOM_e_ of the Ag/MXene composites was obtained by fitting the datapoints to Equation 1.

## Discussion

### Reported data for sheet resistance and transmittance

**Figure 1.**
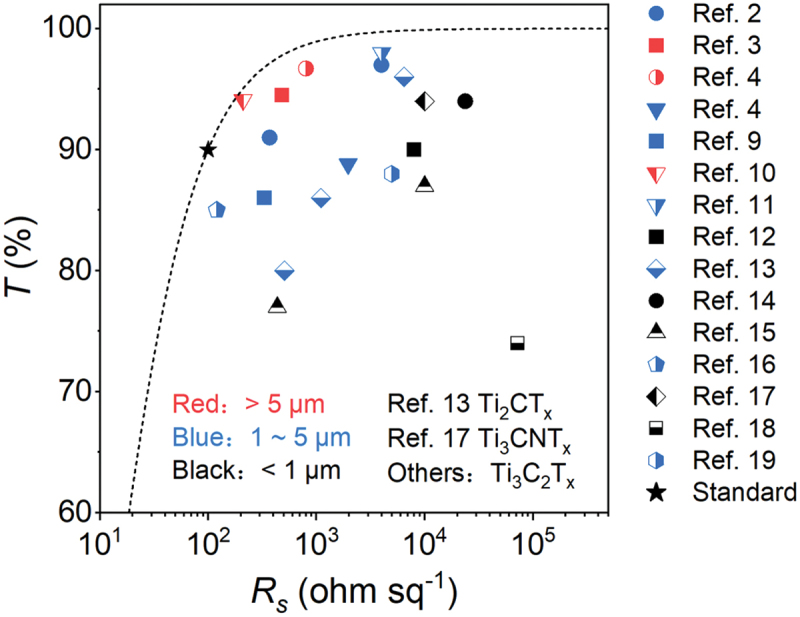
Transmittance (*T*) and sheet resistance (*R*_*s*_) data from literature reports [2–4,9–19]. Data are divided into 3 categories (MXene flake sizes of <1 μm, 1–5 μm, >5 μm). The Black star is the lowest industrial standard value for transparent electrodes.

The reported data for the *T* and *R*_*s*_ of MXene-based TCEs show a significant variation, ranging from high-conductivity to high-resistance films, especially in the high transparency (*T* > 80%) region. We conduct a comprehensive analysis of over 20 studies on MXene-based TCEs, including Ti_3_C_2_T_*x*_, Ti_2_CT_*x*_, Ti_3_CNT_*x*_, focusing on *R*_*s*_ and *T* [2–4,9–19]. Data are extracted from these studies and plotted to show *T* as a function of *R*_*s*_, as depicted in Figure 1. The results reveal that *R*_*s*_ varies by 2 ~ 3 orders of magnitude, from approximately 400 Ω sq^−1^ to around 40 KΩ sq^−1^, even within similar transparency ranges. This underscores the need to investigate the differences among various MXene-based TCEs and identify the key factors that limit their optoelectronic performance.

### Calculated conductivity ratio for published data

**Figure 2.**
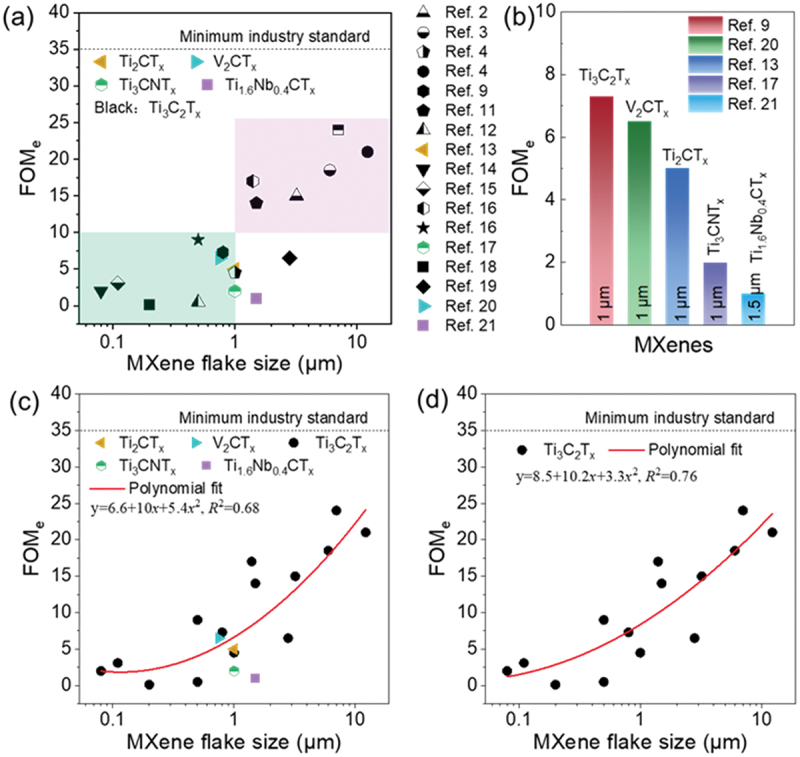
(a) FOM_e_ values of various MXenes as a function of flake sizes. The FOM_e_ ($\sigma_{\mathrm{DC}}$/$\sigma_{\mathrm{op}}$) values are extracted or fitted from *T* and *R*_*s*_ data reported in the literature. (b) Comparison of figure of merit (FOM_e_) of different-type MXene at similar flake sizes. (c) Fitting curve of FOM_e_ value and flake sizes from Ti_3_C_2_T_*x*_ dataset. (d) Fitting curve of FOM_e_ value and flake sizes from various MXene datasets.

We plotted the figure of merit (FOM_e_) values and MXene flake sizes from the same data set. For each system, we generally extract the average or highest value, as shown in Figure 2a. It is evident that there is a clear correlation between the FOM_e_ of these TCEs and the flake sizes, larger MXene flakes exhibit superior optoelectronic properties compared to those of smaller ones. We define flake size of ~1 μm as the cutoff standard; flakes (several hundred nanometers) smaller than this are typically produced through sonication. Interestingly, the graph can be divided into two sections, MXene films with flake sizes below 1 μm never show $\sigma_{\mathrm{DC}}$/$\sigma_{\mathrm{op}}$ values larger than 10, while the FOM_e_ value for MXene films with flake sizes larger than 1 μm tend to exceed 10 with ease. For instance, a maximum FOM_e_ of ~24 was achieved in Ti_3_C_2_T_*x*_ TCEs with flake size of ~7 μm. Among various types of MXene with similar flake sizes, Ti_3_C_2_T_*x*_ exhibits the highest FOM_e_ compared to other MXene types, indicating that this type of MXene is the choice of option (Figure 2b) [9,13,17,20,21]. Therefore, Ti_3_C_2_T_*x*_ is selected as the representative material for further analysis of the optoelectronic properties of TCEs. We performed polynomial fitting (other fitting methods yielding more unrealistic results) for the relationship between Ti_3_C_2_T_*x*_ flake sizes and FOM_e_ (Figure 2c), as well as for the sizes of various types of MXenes and FOM_e_ (Figure 2d). The coefficients of determination (*R*^2^) were 0.76 and 0.65, respectively. Although the R^2^ values are not particularly high, the fitted curves still capture the trends in the experimental data, and the model parameters are consistent with practical relevance. This suggests that increasing flake size appears to be an effective and feasible approach, particularly for undoped pure MXene.

**Figure 3.**
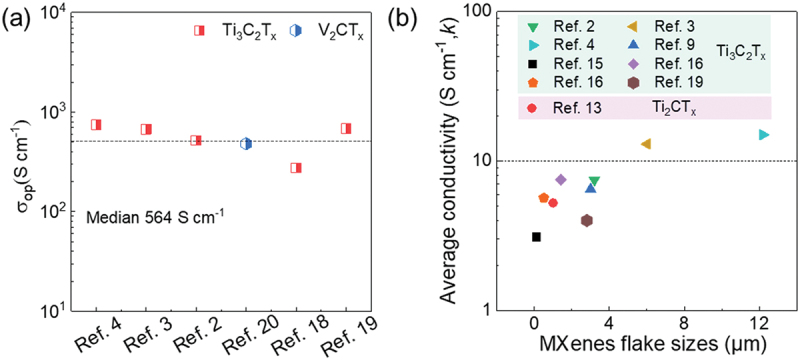
The optical conductivity (a) and DC conductivity (b) extracted from the literature.

We further analyze whether variations in $\sigma_{\mathrm{DC}}$ or $\sigma_{\mathrm{op}}$ are responsible for the wide range of FOM_e_ values observed. Only a few reports provide the thickness of the TCEs, making it impossible to calculate $\sigma_{\mathrm{DC}}$ and $\sigma_{\mathrm{op}}$ in many cases. We extract available data and organized it in Figure 3a. The $\sigma_{\mathrm{op}}$ values vary randomly between 275 and 750 S cm^−1^, with a median value of 564 S cm^−1^. This variation depends on the intrinsic characteristics of the material and the number of layers per unit volume. The optical conductivity differences of Ti_3_C_2_T_*x*_ TCEs are further analyzed, with $\sigma_{\mathrm{op}}$ values of 520, 675, 750, 275, 680 S cm^−1^ derived from blade coating, slot-die coating, spin coating, and inkjet printing, gravure printing, respectively [2–4,18,19]. Guo et al. demonstrated that TCEs fabricated using blade coating or slot-die coating technologies exhibit higher orientation compared to those produced by spin-coating, as observed in surface morphology [3,4]. We speculate that the variation in $\sigma_{\mathrm{op}}$ is due to morphological differences between the films, such as surface roughness, free volume, and compactness. Although slight differences in $\sigma_{\mathrm{op}}$ between Ti_3_C_2_T_*x*_ and V_2_CT_*x*_ (481 S cm^−1^) [20] are reported here, we consider this difference to fall within an acceptable fluctuation range due to the limited data available. However, the average DC conductivity ($\sigma_{\mathrm{DC}}$) of the TCEs varies significantly, ranging from 3,092 to 15,000 S cm^−1^ (Figure 3b). This suggests that the variation in FOM_e_ is primarily influenced by changes in $\sigma_{\mathrm{DC}}$.

In MXene TCEs, charge carriers migrate along the direction with the lowest energy barrier within a single flake, then overcome the interflake tunneling barriers to jump onto another flake. Therefore, the total resistance (*R*_*tot*_) of the TCEs consists of in-plane resistance which represents the inherent resistance (*R*_*inh*_) of the MXene flakes, and interflake resistance (*R*_*int*_) which relates to the quantity of interflake junctions in the horizontal direction and intercalants in the vertical direction [22]. Here, the inherent resistance and the residual Li^+^ content are mainly determined by the etching and delamination process. Previous studies have shown that the LiF/HCl etching route results in fewer defects on individual flakes compared to HF etching [23], and reducing the residual Li^+^ between layers can improve conductivity [24]. In all cases, the delaminated MXene used for TCE preparation was obtained via conventional HF or LiF/HCl etching routes.

Additionally, the significant difference in conductivity of Ti_3_C_2_T_*x*_-based TCEs reflects variations in the number of interflake tunneling barriers. The number of these barriers depends on the flake sizes and the degree of aggregation or delamination. In films prepared from adequately delaminated, large-sized MXene flakes, there are relatively few interflake barriers. In contrast, as the flake sizes decrease, the number of boundaries significantly increases. This explains why the FOM_e_ of large-sized MXene films appears in the upper region of Figure 2a, while the FOM_e_ of small-sized films is found in the lower region. Thus, empirically, the FOM_e_ of TCEs can be expressed as FOM_e_ ∝ $\sigma_{\mathrm{DC}}$, or FOM_e_ ∝ *R*_*tot*_^−1^= (*R*_*inh*_ +*R*_*int*_)^−1^. Here, *R*_*tot*_ ≈ *R*_*inh*_, when the flake size approaches infinity, meaning that $\sigma_{\mathrm{DC}}$ and FOM_e_ values become dependent of the inherent conductivity. Consequently, to achieve the highest performance, we should prepare MXenes with few defects such as pinholes or micropores in the in-plane flakes and smooth edges.

## Limiting values of $\sigma_{\mathrm{DC}}$/$\sigma_{\mathrm{op}}$

The $\sigma_{\mathrm{DC}}$ and $\sigma_{\mathrm{DC}}$/$\sigma_{\mathrm{op}}$ of TCEs are influenced by MXene types (intrinsic resistance), flake sizes, and the degree of delamination (interflake resistance). We anticipate that $\sigma_{\mathrm{DC}}$ can be maximized by fabricating large-sized, highly delaminated MXene flakes. In our previous work, we prepared predominantly large-sized Ti_3_C_2_T_*x*_ flakes (12.2 μm) with a narrow size distribution [4]. Films from these flakes exhibit the highest FOM_e_ of 29, with *R*_*s*_ = 77 Ω sq^−1^ at *T* = 83.4%. The TCE demonstrates a high $\sigma_{\mathrm{DC}}$ of 19,325 S cm^−1^, which, to our knowledge, is the highest reported value from conventional etching methods (HF or HCl/LiF) for Ti_3_AlC_2_. Unlike liquid-exfoliated graphene flakes, delaminated MXene flakes are obtained by etching the as-received MAX phase using hydrofluoric (HF) or HCl/HF etching methods, or by in situ HF formation methods (such as LiF/HCl with or without intercalants). These methods pose challenges in obtaining large, delaminated Ti_3_C_2_T_*x*_ flakes (larger than 10 μm) with a broad size distribution due to the inherent limitations of the synthesis process. Gogotsi et al. have reported Ti_3_C_2_T_*x*_ with an average lateral size of 14 μm and a maximum size up to 40 μm, albeit in small quantities [25]. Notably, to meet the minimum industrial standard of FOM_e_ > 35, maximizing the conductivity of TCEs and minimizing interfacial resistance become critical. Assuming the use of ultralarge Ti_3_C_2_T_*x*_ flakes (up to 40 μm) to fabricate TCEs via the slot-die coating process (with an optical conductivity of 675 S cm^−1^), applying the formula FOM_e_ = σ_DC_/σ_op_, it can be inferred that the DC conductivity of TCEs needs to exceed 23,625 S cm^−1^, which is evidently achievable. This inference is supported by two key findings, (1) MXene flakes with an average size of 12.2 μm have already achieved a maximum DC conductivity of approximately 20,000 S cm^−1^ [4], demonstrating the potential of larger flakes in reducing interflake resistance and enhancing conductivity. (2) Zeraati et al. reported achieving a DC conductivity of 24,000 S cm^−1^ using Ti_3_C_2_T_*x*_ flakes with an average size of only 1.8 μm via the evaporated-nitrogen minimally intensive layer delamination (EN-MILD) method [26]. We believe that preparing large-sized Ti_3_C_2_T_*x*_ flakes through the EN-MILD method will significantly reduce interflake resistance, which could lead to enhanced DC conductivity and improved σ_DC_/σ_op_ values, thus enabling superior performance in TCEs applications.

## Surpassing limits through integration with metal mesh

Obviously, the pure MXene TCEs based on continuous metallic films give a practical maximum FOM_e_ of 24, which falls short of the required standard for advanced optoelectronic devices such as the touch panels. Unless ultralarge MXene nanosheets are prepared using special, complex processes as described above (which typically have low yield), this approach, although theoretically feasible, faces significant challenges in practical implementation. To beat the practical limitations, one should revolutionize the TCE architecture by replacing continuous films with mesh structures to substantially improve *T* without affecting *R*_s_. As such, advanced TCEs can be fabricated using conventional small MXene nanosheets. This is especially true when the mesh is rationally designed, allowing more light to pass through instead of absorbed by the MXene flakes, leading to much increased *T*. Unfortunately, creating MXene mesh on substrates inevitably decreases the uniformity of corresponding TCEs. To counter such a negative effect, depositing metal grids onto the uniform MXene film to create a metal grid/MXene structure should result in much lower *R*_*s*_ at much higher *T*, along with uniform electrical, optical and thermal conductivity. As such, we break the FOM_e_ limitation of MXene TCEs by fabricating metal grid/MXene hybrid TCEs, making them a promising alternative to fragile ITO in applications.

**Figure 4.**
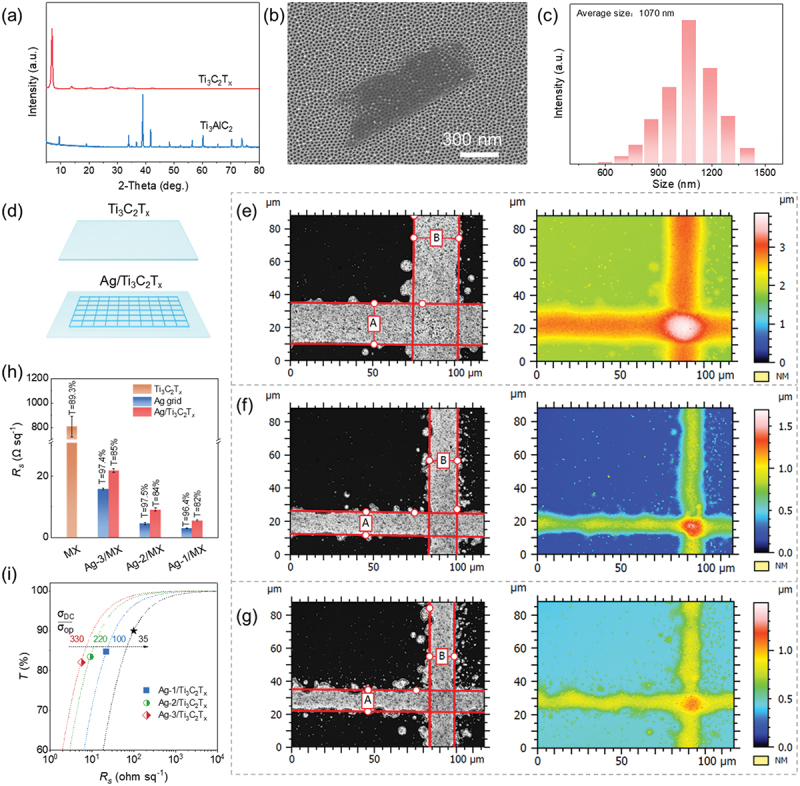
(a, b, c) XRD patterns, SEM image, and particle size distribution of the Ti_3_C_2_T_*x*_ flakes, respectively. (d) Scheme of the printed grids on the MXene TCE. (e, f, g) the width and height of Ag lines under printing speed 6, 9, 12 mm s^−1^, respectively. Left side: width, Right side: height. (h) Sheet resistance and transmittance of Ti_3_C_2_T_*x*_, Ag grid and Ag-grid/MX hybrid TCEs. Ti_3_C_2_T_*x*_ flakes size is about 1 μm. Ag grid was printed onto the Ti_3_C_2_T_*x*_ TCE. (i) The relationship of Ag-grid/MX hybrid TCEs between *T* with *R*_*s*_, also included are fitting curves according to Equation 1. the Black star is the lowest industrial standard value for transparent electrodes.

The Ag grids/Ti_3_C_2_T_*x*_ (Ag/MX) hybrid TCEs were accomplished by digitally controlled aerosol jet printing of Ag ink onto dried Ti_3_C_2_T_*x*_ TCEs. The XRD patterns and size distribution of the Ti_3_C_2_T_*x*_ flakes are shown in Figure 4(a,b,c) with the flake size being approximately 1 µm. Here, these uniform Ti_3_C_2_T_*x*_ films were deposited onto a glass substrate using the slot-die coating technique, with the details provided in our previous work [3]. The hybrid TCEs were manufactured with an area of 5 × 5 cm^2^, featuring a line pitch of 1 mm in both vertical and horizontal directions, schematics shown in Figure 4d. To control the width and height of the Ag lines, we adjusted the printing speed, resulting in three hybrid TCEs variants with average Ag line dimensions of 26.2/0.839 μm, 15.4/0.497 μm, and 14/0.298 μm for width/height, respectively. These variants are labeled as Ag-1/MX, Ag-2/MX, and Ag-3/MX (Figure 4e,f,g). The Ag-1, Ag-2, and Ag-3 grids demonstrated high transparency of 96.4%, 97.5%, and 97.4%, with corresponding sheet resistances of 3.1, 4.6, and 15.8 Ω sq^−1^, respectively. When these Ag-1, Ag-2, and Ag-3 grids were deposited onto Ti_3_C_2_T_*x*_ TCEs (*T* = 89%), the transparency of the hybrid TCEs decreased to 82.1%, 83.5%, and 84.7%, with corresponding sheet resistances of 5.6, 9.2, and 21.9 Ω sq^−1^, respectively (Figure 4h). This indicates that the transmittance of the hybrid TCEs primarily depends on the Ti_3_C_2_T_*x*_ TCE at the same line pitch of the Ag grid. As shown in Figure 4i, the FOM_e_ values for Ag-1/MX, Ag-2/MX, and Ag-3/MX were 100, 220, and 330, respectively, representing a significant improvement compared to pure continuous MXene TCEs. To meet the requirements of industrial applications for TCEs that are made from small-sized MXene nanosheets produced using conventional synthesis methods, without relying on strict processes to produce high-quality large-sized MXene nanosheets (which typically have low yield), depositing Ag or other metal mesh structures onto MXenes for hybrid TCE fabrication is a promising design strategy, which could enable MXenes to replace ITO in the future.

## Conclusion

In this work, we analyzed both the potential of MXene-based transparent conductive electrodes (TCEs) for industrial applications and their theoretical limitations of FOM_e_, while also developing hybrid Ag-grid/MXene TCEs to overcome these challenges. While pure MXene continuous films show promising conductivity and transparency, their figure of merit (FOM_e_) saturates at around 29, which is far below the minimum industrial requirement of 35. The limitations of MXene-based transparent conductive electrodes (TCEs) arise from the inherent properties of MXene flakes, such as their type, size, yield, and challenges in achieving high electrical conductivity and low optical conductivity. These factors lead to insufficient technical specifications and pose challenges for large-scale production. Here, key challenges include the difficulty in obtaining large, defect-free flakes and the inability to achieve the required balance of conductivity and transparency. Hybridizing MXene with metal mesh structures is the future for TCEs, as this strategy significantly improves the FOM_e_, providing a scalable solution to meet industrial standards and enabling MXene-based TCEs to replace ITO in practical applications.
